# Establishment and Validation of Whole-Cell Based Fluorescence Assays to Identify Anti-Mycobacterial Compounds Using the *Acanthamoeba castellanii* - *Mycobacterium marinum* Host-Pathogen System

**DOI:** 10.1371/journal.pone.0087834

**Published:** 2014-01-31

**Authors:** Sébastien Kicka, Valentin Trofimov, Christopher Harrison, Hajer Ouertatani-Sakouhi, John McKinney, Leonardo Scapozza, Hubert Hilbi, Pierre Cosson, Thierry Soldati

**Affiliations:** 1 Department of Biochemistry, University of Geneva, Geneva, Switzerland; 2 Max von Pettenkofer Institute, Ludwig Maximilians University, Munich, Germany; 3 Department of Cell Physiology and Metabolism, Faculty of Medicine, University of Geneva, Geneva, Switzerland; 4 Global Health Institute, Swiss Federal Institute of Technology in Lausanne (EPFL), Lausanne, Switzerland; 5 Pharmaceutical Biochemistry Group, School of Pharmaceutical Sciences, EPGL, University of Geneva, Switzerland; University of Lausanne, Switzerland

## Abstract

Tuberculosis is considered to be one of the world’s deadliest disease with 2 million deaths each year. The need for new antitubercular drugs is further exacerbated by the emergence of drug-resistance strains. Despite multiple recent efforts, the majority of the hits discovered by traditional target-based screening showed low efficiency *in vivo*. Therefore, there is heightened demand for whole-cell based approaches directly using host-pathogen systems. The phenotypic host-pathogen assay described here is based on the monitoring of GFP-expressing *Mycobacterium marinum* during infection of the amoeba *Acanthamoeba castellanii*. The assay showed straight-forward medium-throughput scalability, robustness and ease of manipulation, demonstrating its qualities as an efficient compound screening system. Validation with a series of known antitubercular compounds highlighted the advantages of the assay in comparison to previously published macrophage-*Mycobacterium tuberculosis-*based screening systems. Combination with secondary growth assays based on either GFP-expressing *D. discoideum* or *M. marinum* allowed us to further fine-tune compound characterization by distinguishing and quantifying growth inhibition, cytotoxic properties and antibiotic activities of the compounds. The simple and relatively low cost system described here is most suitable to detect anti-infective compounds, whether they present antibiotic activities or not, in which case they might exert anti-virulence or host defense boosting activities, both of which are largely overlooked by classical screening approaches.

## Introduction

### Tuberculosis, a Serious Health Threat

The negative impact that tuberculosis (TB) has on human health is hard to overestimate. Over one third of the world population is infected by bacteria of the *Mycobacterium tuberculosis* (Mtb) complex. Each year two million TB-related deaths are registered with 8 million newly infected people [1]. Despite the efforts of modern therapeutics, in nine of ten cases, Mtb manages to persist throughout the lifetime causing the risk of reinfection and reescalation of the disease [2].

The hallmark of TB is the formation of granuloma, well-organized multicellular structures primarily composed of mature macrophages and T-lymphocytes. Macrophages often develop into multinucleated giant cells and epitheloid cells. Granulomas also contain dendritic cells, neutrophils, NK-cells, fibroblasts and B-lymphocytes and are surrounded by a fibrous cuff. In addition, epithelial cells surrounding granulomas are proposed to participate in its formation. It is generally assumed that the granuloma is a host-defensive structure that sequesters and eradicates pathogenic bacteria. Although there is evidence of healed and often sterile granuloma among certain TB patients, recent findings indicated that Mtb employs a distinct mechanism of proliferation via granulomas [22]. Mtb mostly replicates in alveolar macrophages but can also be found in dendritic cells, adipocytes and type II alveolar pneumocytes [3]–[6].

Pathogenic mycobacteria, such as Mtb and other mycobacteria of the tuberculosis complex, but also *Mycobacterium leprae, Mycobacterium marinum* and *Mycobacterium avium* are able to manipulate a variety of processes including membrane trafficking [3], [7], autophagy [8], [9], signaling [10] and apoptosis [11], [12]. These manipulations of its host allow the bacteria to hijack the phagosome and prevent major steps of its maturation by performing rapid exclusion of the vacuolar H-ATPases [13], inhibiting the action of signalling lipids [14], [15] and proteins [16], [17] involved in phagosome maturation.

In order to find a way to counteract TB infection, considerable research efforts focus on a mechanistic study of mycobacterial virulence factors. One of them is encoded by the RD1 locus, which was first discovered by investigating the genome deletions in the attenuated *Mycobacterium bovis* BCG vaccine strain. Studies performed also with *M. marinum* found it to be the main virulence determinant [18], [19]. The locus encodes a type 7 secretion system, called ESX-1 system [18]. It was shown that RD1 mutants are less effective in arresting phagosome maturation and are attenuated in infection dissemination [18], [20]–[23].

### TB Treatment, Search for New Drugs

The standard treatment for tuberculosis uses a combination of antitubercular compounds for six months or longer. The necessity of extensive treatment was elaborated after a long period of trial and error. It is now clear that noncompliance with the treatment, short-term and relaxed therapy regimens result in the emergence of drug resistant strains. The situation has escalated even further due to emergence of multi-drug resistant (MDR) strains and, finally, extensively drug resistant (XDR) strains, and more recently some totally drug resistant strains have been described [24]. As a consequence, the WHO reviewed the strategy to fight TB infections, leading to the establishment of “directly observed treatment short course” (DOTS) (WHO report 2011).

The newest drug for TB treatment is 30 years old, and the previously very effective streptomycin lost its efficiency against *M. tuberculosis* and is no longer used for therapy. Therefore, the need for new drugs has become obvious. Several reasons underlie the lack of new drugs, such as the difficulty to identify compounds that penetrate mycobacteria, because of the low permeability of the mycolate-rich cell wall or because of the low metabolic and growth rates reflected by their 24–36 hours doubling time. In addition, conventional screening approaches usually favour the search for bactericidal compounds while at the same time neglecting host-pathogen interactions.

Despite the challenges mentioned above, several drug candidates are currently under development and have a good chance to enter the market. Promising approaches for drug development include targeting synthesis of lipids as nutrients [25], [26] and synthesis of mycolic acids as major components of the cell wall [27]. In the last decade, researchers have identified compounds that kill dormant bacteria by intracellular NO release, such as the bicyclic nitroimidazoles, PA-824 [28], and OPC-67683, as well as compounds that affect ATP-synthesis such as TMC207 [29] and nitrofuranylamide compounds with so far unknown mode of action [30]. Some screens revealed prodrugs that are activated by the metabolism of the host cell, such as nitroimidazopyran [31]. Potentially interesting compounds also include heterocyclic aldehydes [32], oxazole- and oxazoline-containing compounds that target iron uptake [33], and rhodanine derivatives that target the dihydrolipoamide acyltransferase [34].

### Whole-cell Based Screening, a Promising Alternative to Target-based Approaches

Standard target-based approaches identified compounds that showed very high attrition rates and low numbers of validated hits against the intact live bacterium and in infection systems [35], [36]. Meanwhile, a broad spectrum of new tools has become available [37]. A new trend has emerged: phenotypic screens in a whole-cell infection system [38], [39]. Whole-cell screens are a promising method to provide lead-structures and identify new targets. Unlike target-based approaches they fulfill *in vivo* criteria such as membrane permeability and a higher activity against mycobacteria than host cells. However, whole-cell based assays typically do not easily reveal the mechanism of action. Additional mechanistic studies and rounds of structure-activity relationship investigations are required.

Establishing alternative methods to target-based screening may improve the chances to discover new sets of drugs that could be competitive with current drugs, shorten the duration of treatment, avoid significant drug-drug interactions, and successfully deal with MDR and XDR Mtb strains. The ability of whole-cell screens to detect host response *in situ* makes it possible to reveal not only antibiotic activities, but also anti-infective drugs. Such compounds target infection-specific biological processes, and therefore, significantly reduce the risk of acquiring resistance. A proof of feasibility for the identification of such active compounds was established in a few recent studies (reviewed in [40]). For *M. tuberculosis,* the list contains inhibitors of iron metabolism [41] and compounds targeting resistance to oxidative stress [34]. Moreover, whole cell-based approaches allow detection of compounds that increase the activity of natural, host-specific innate immune defense mechanisms. This opens the possibility of discovering compounds capable of helping the host cell deal with a broad range of pathogens. For example, the cellular pool of kinases and phosphatases was shown to be the targets of defense-boosting compounds [39], [42]–[44].

### *Mycobacterium marinum* as a Pathogen Model for Drug Screening Purposes

As mentioned above, despite the fact that many screens resulted in the discovery of promising antimycobacterial compounds, overall screening for anti-Mtb drugs remains ineffective [29], [30], raising the demand for new strategies, including the use of new and more cost-efficient host-pathogen models.

*M. marinum,* the closest relative of Mtb in the tuberculosis complex, is an attractive alternative model. *M. marinum* is a fish and frog pathogen which establishes an infection similar to human tuberculosis [34]. Bacterial growth temperature is optimal at 30°C, rendering it less dangerous for humans, as it is only capable to establish superficial skin lesions [45]. Moreover its doubling time of eight hours is much shorter than that of Mtb and *M. bovis BCG,* which in turn improves the speed of detection of antimycobacterial effects. For *M. marinum* the mechanisms of phagosome maturation arrest, as well as the activity of many virulence genes are very similar to *M. tuberculosis* [20], [46]. *M. marinum* readily escapes its vacuole [47], but the efficiency and relevance of this process for Mtb is still debated [48]. The high degree of functional conservation in virulence genes supports the theory that ancient mycobacterial precursors developed the mechanisms of pathogenesis against phagocytic protozoa and that mycobacteria are now using them to hijack animal immune phagocytes [49]. Indeed it has been shown that free-living amoebae can be an environmental reservoir for pathogenic bacteria such as *M. avium*, *M. marinum* and even Mtb [50].

For *M. marinum,* well-developed genetic and cellular biology tools are available. These features render *M. marinum* extremely useful for the investigation of the mode of action of antitubercular compounds and for the validation with the *M. tuberculosis* model [51].

### Amoebae Host Systems for Drug Screening

Among whole-cell based assays the usage of unicellular hosts is advantageous because of the ease of cultivation and manipulation important in high-throughput screening. Although protozoa do not engage in complex multicellular interactions, the high degree of conservation of innate immune defense mechanisms renders them attractive alternative systems for experimental infection studies. Within the host one can target multiple pathways involved at different stages of the infection, including endosomal trafficking during phagocytosis of the bacteria, the autophagy pathway in the form of xenophagy, ion-pumps recruitment involved in bacteria degradation during phagosome maturation, kinases and phosphatases signaling that affect the course of infection (reviewed in [49], [52], [53]).

Since the primary target of Mtb is macrophages, amoebae that are also professional phagocytes, are a rational choice to study host-pathogen interactions. Amoebae offer a well-balanced compromise between the natural complexity of the system on one side and ease of manipulation and cultivation on the other. Amoebae and macrophages possess a high degree of functional conservation in defense mechanisms against infection [54]. Many species of amoebae serve as a natural reservoir and a training field for pathogens.

*Acanthamoeba* are a particularly promising genus of amoebae for screening purposes. Its environmental niches include soil, air and fresh water. Unlike *Dictyostelium discoideum,* a soil-inhabiting social amoeba that is another popular protozoan model, *Acanthamoeba* does not undergo a multicellular developmental phase, which probably renders them less sensitive to the stress factors inevitable in screening processes. *Acanthamoeba* is considered to be a natural carrier for many mycobacteria species [55], for example it was shown that 25 mycobacteria species, including non-tuberculous mycobacteria, can infect both trophozoites and cysts of *Acanthamoeba polyphaga* [56]–[58]. Moreover, intracellular *M. avium* within *Acanthamoeba castellanii* showed increased resistance to bactericidal compounds such as rifabutin, compared to growth within macrophages [56], [59]. The ability to protect from antitubercular drugs may serve as an additional *in vivo* filter to subtract false-positive hits of drug screening. On the other hand, the *D. discoideum* model system has its own unique advantages, particularly a complete set of genetic tools that are extremely useful for the determination of mechanisms of action. Together with a fully sequenced and annotated haploid genome, *D. discoideum* is amenable to forward and reverse genetics. Its simplicity of cultivation makes it easily biochemically tractable. *D. discoideum* also allows excellent real-time live imaging. Taken together, both amoeba genera are useful models, each having its advantages depending on the purpose of the experiments.

In the present study we have established the *A. castellanii* – *M. marinum* host-pathogen system as a robust compound screening and validation system. Together with secondary assays using *D. discoideum* and *M. marinum*, it shows excellent promise to identify novel antitubercular hits.

## Results

### A Fluorescence- and Cell-based Assay to Measure Intracellular Mycobacterial Growth in *A. castellanii*

**Figure 1:**
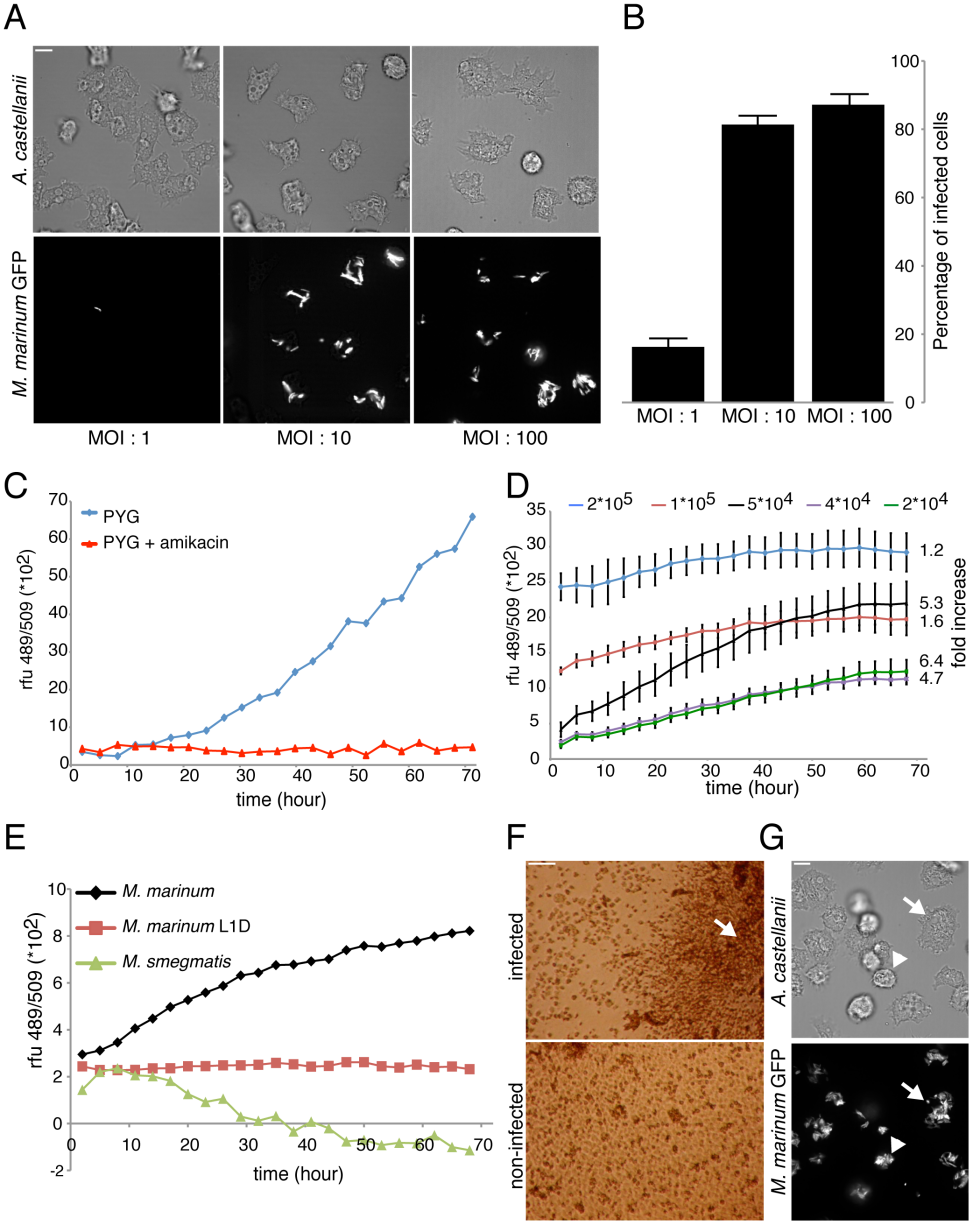
Mycobacteria infection of *A. castellanii*. **A.** Confocal (top) and brightfield (bottom) pictures of *A. castellanii* infected with GFP-expressing *M. marinum* at different MOI. Scale bar, 10 µm. **B.** Corresponding percentage of infected cells under the three MOI conditions, error bars represent the standard deviation from technical replicates (three microscopy fields and at least forty counted cells) of one representative experiment. **C.** Growth kinetics of *M. marinum* msp12::GFP in PYG medium supplemented with 10 µM amikacin, representative experiment from a series with similar outcome. **D.** Growth kinetics of GFP-expressing *M. marinum* within *A. castellanii* plated at different densities, measured by total fluorescence intensity. The standard deviation derives from the technical mean of eight microwells. The values on the right represent the fold fluorescence increase between 2 and 68 hours post infection. **E.** Representative experiment of growth kinetics of GFP-expressing *M. smegmatis*, *M. marinum* WT and L1D mutant strains within *A. castellanii,* measured by fluorescence intensity. **F.** Brightfield microscopy of the cells at the bottom of a microwell. Infected cells at 3 DPI under control conditions and non-infected cells. Scale bar is 100 µm. **G.** Phase contrast (top) and confocal (bottom) pictures of *A. castellanii* infected cells with GFP-expressing *M. marinum* at three days post-infection. Infected giants cells (arrow), and some dead cells (arrowhead) are observed. Scale bar, 10 µm.

In this study, we present a fast and easy approach to identify compounds with anti-infective properties in a cellular host context. *M. marinum* is able to replicate efficiently in the free-living fresh water amoeba *A. castellanii* [60]. We therefore used *A. castellanii* to monitor intracellular growth of *M. marinum* using a fluorescence-based assay. *A. castellanii* was chosen instead of our *D. discoideum* model due to its ‘macrophage-like’ size that allows a higher level of bacteria uptake, easily detectable with our fluorescence plate reader. The protocol established uses an optimized multiplicity of infection (MOI) and is based on synchronous and homogeneous bacterial phagocytosis. Spinoculation of mycobacteria on top of a cell monolayer close to confluency maximizes host-bacteria contact and subsequent uptake and thus guarantees high reproducibility of the infection course. The percentage of infected cells at time zero and the average number of bacteria per cell is a function of the MOI (Figure 1A). Using an MOI of 10∶1 ensures that a majority of cells (≈ 80%) are infected (Figure 1B) with approximately 1 to 5 bacteria per cell, and thus, this condition became our infection standard. Then, excess extracellular bacteria were carefully removed by washing with PYG medium, prior to testing compounds of interest on infected cells. [61]–[63]Finally, infected cells were resuspended in PYG medium containing amikacin to prevent extracellular growth of bacteria [64]. Using 10 µM amikacin prevented bacteria proliferation in PYG medium for at least three days (Figure 1C).

In order to adapt the monitoring of infection to a larger scale, a fluorescence plate reader assay was optimized for the 96-well plate format. Use of mycobacteria strains expressing fluorescent reporters has recently been validated for the quantitative measurement of bacterial mass, as an alternative to c.f.u. counting, both for microscopy on live and fixed cells and organisms, as well as higher throughput methods such as microwell-plate readers [61]–[63]. The fluorescent *M. marinum* msp12::GFP strain [65] gave us the most robust readout to quantitate the increase in bacterial numbers, but other fluorescent and bioluminescent reporters can also be used [66]. As shown in figure 1D, the plating density of initially infected cells (obtained with an MOI 10∶1) was a parameter that greatly impacted intracellular bacterial growth. As indicated at the right of the graph, the fluorescence fold increase at 3 days post infection (DPI) was low at high cell density (1.2 and 1.6 for 2*10^5^ and 1*10^5^ cells/well, respectively) and bacterial growth kinetics reached a plateau after 30–40 hours post infection (HPI). In contrast, lower densities that allowed host cells to grow for at least two days before reaching confluency, resulted in a higher bacterial expansion (4–6 fold increase with 2 to 5*10^4^ cells/well). Therefore, we decided to plate between 1 and 5*10^4^ infected cells in each well of the 96-well plate.

We validated our assay by monitoring *A. castellanii* infection with the non-pathogenic mycobacterium, *Mycobacterium smegmatis*, and an avirulent mutant, *M. marinum*-L1D [65]. As presented in figure 1E, the total fluorescence of GFP-*M. smegmatis* decreased over time, indicating that the bacteria are killed and digested by the amoebae. In addition, similarly to its fate in zebrafish and macrophages, the *M. marinum*-L1D mutant was not able to replicate in *A. castellanii*. Similar results have been reported using the *D. discoideum* host [47]. However, *M. marinum*-L1D’s fluorescence remained stable, indicating that *A. castellanii* appears unable to fully digest this strongly attenuated *M. marinum* strain.

Under our conditions, *A. castellanii* infection with *M. marinum* appeared to severely decrease the growth and/or survival of the amoebae. Inspection of the wells by phase contrast microscopy showed that infected *A. castellanii* cells did not reach maximal confluency at 3 DPI, concomitantly with a notable accumulation of extracellular bacteria at the well centre (Figure 1F, arrow). Further inspections showed that heavily infected and dead amoebae, as well as abnormal giant cells are often observed during the late phase of infection (Figure 1G, arrowhead and arrow, respectively). Lethality induced by *M. marinum* infection has been reported in many animal systems such as the *Drosophila* larvae [67], leopard frog [68], and in macrophages [69]. Amoeba lysis has also been mentioned as a result of infection with other intracellular pathogens, such as *Legionella pneumophila* [70].

### Drug Validation

**Figure 2:**
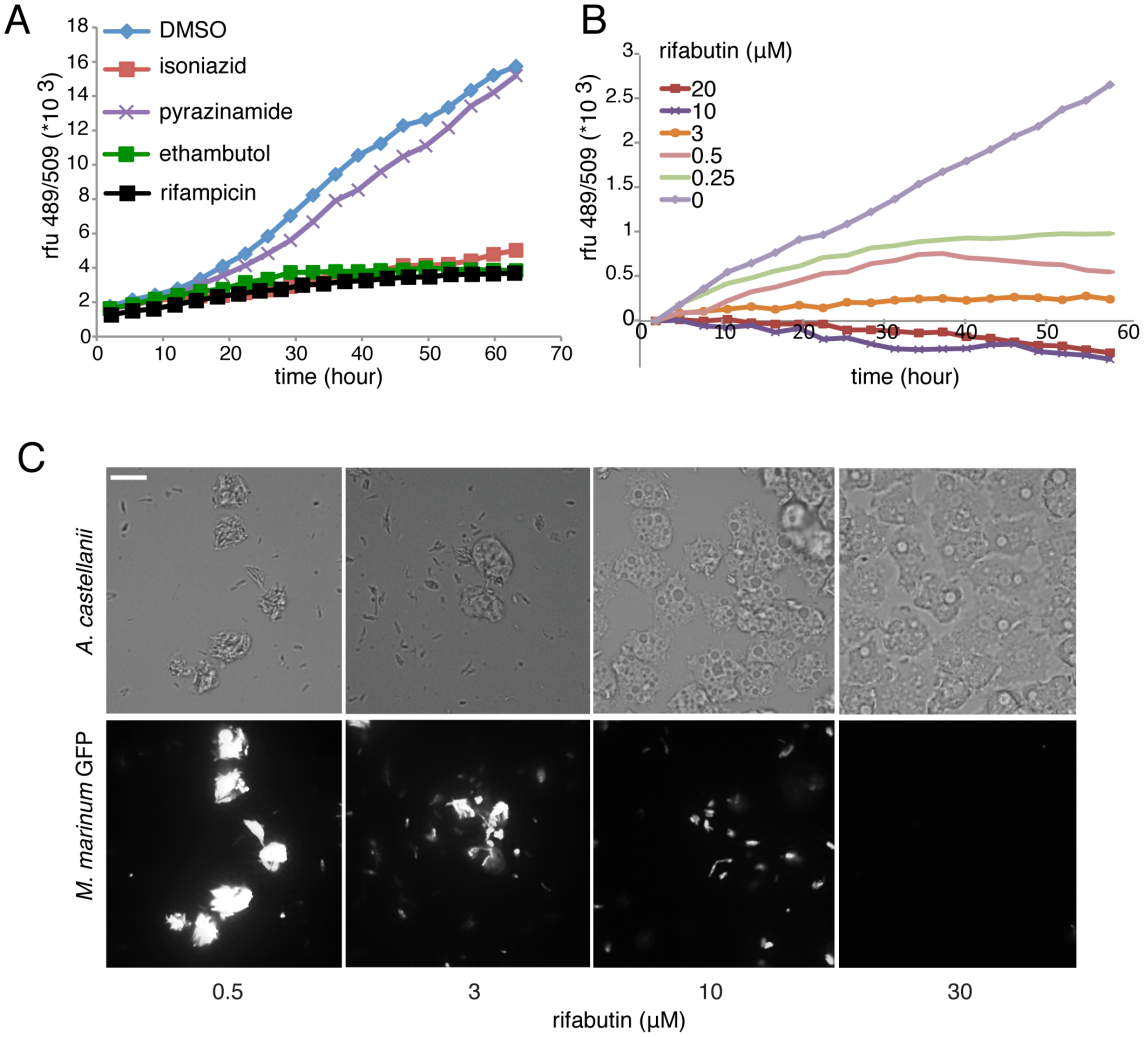
Effect of antibiotics on intracellular growth of *M. marinum* during an infection. **A.** Intracellular growth kinetic of *GFP-expressing M. marinum* measured by fluorescence intensity in the presence of 30 µM of first-line antibiotics. **B.** Intracellular growth kinetics of GFP-expressing *M. marinum* measured by fluorescence intensity, in the presence of different concentrations of rifabutin. A and B are representative experiments from a series with similar outcome. **C.** Effect of rifabutin on *M. marinum* growth during an infection. Brightfield (top) and spinning disc confocal (bottom) imaging of *A. castellanii* infected with GFP-expressing *M. marinum* in the presence of the indicated concentrations of rifabutin, 72 hours post infection.

**Figure 3:**
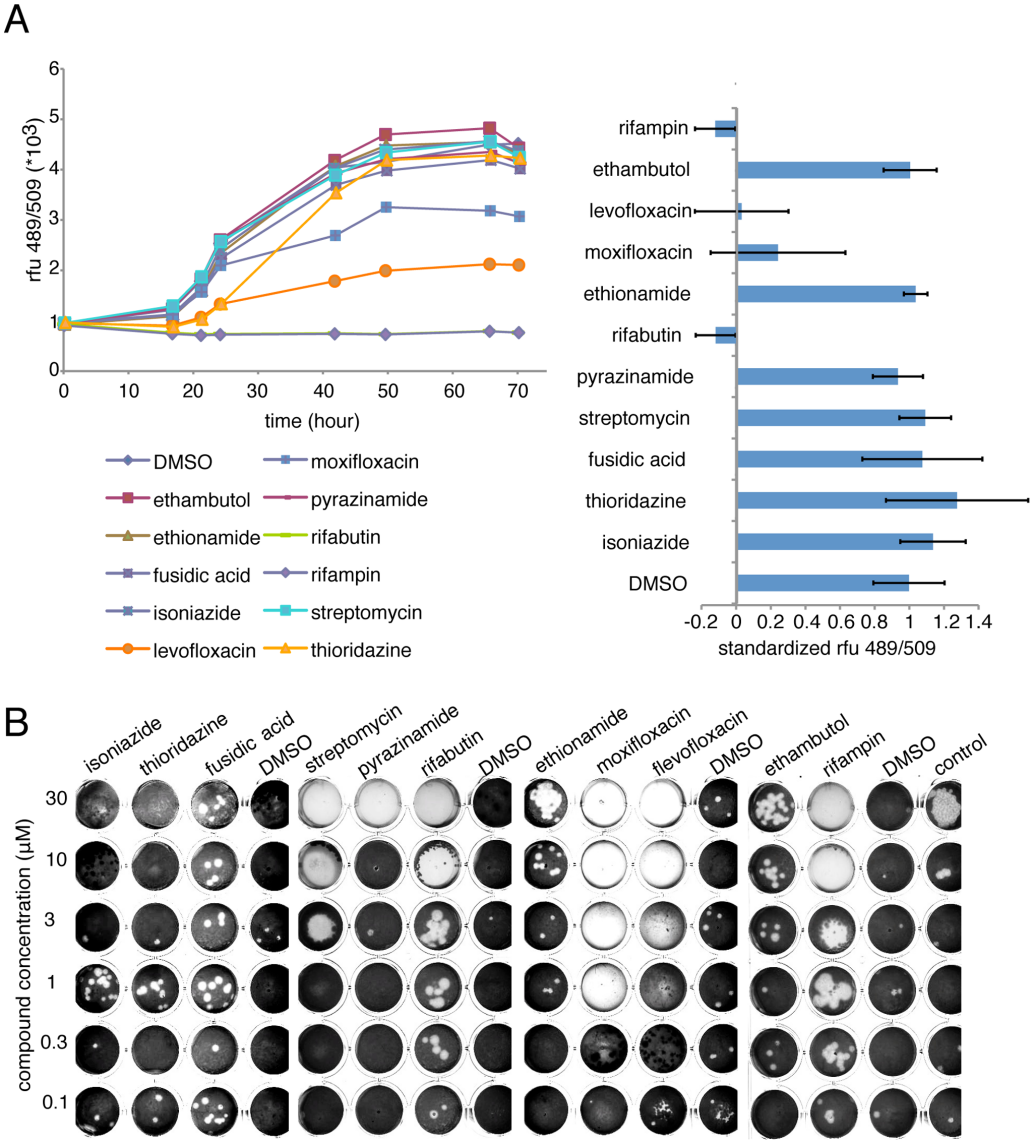
Effect of antibiotics on intracellular growth of *L. pneumophila* during an infection and in a ‘phagocytic plaque assay’. **A. Left,** representative experiment from a series with similar outcome of intracellular growth kinetic of GFP-expressing *L. pneumophila* measured by fluorescence intensity in the presence of 30 µM of antibiotics. **Right**, normalized intracellular bacterial growth (DMSO = 1) in A. castellanii, error bars represent the standard deviation from biological triplicates. **B.** Ability of *D. discoideum* DH1 strain (1000 cells/well) to grow on a bacterial lawn composed of *M. marinum* and *Klebsiella pneumoniae* (2::1 ration) after seven days and in presence of antibiotic compounds.

As a close cousin of *M. tuberculosis*, *M. marinum* is sensitive to most standard antibiotics used to treat tuberculosis, and also used to validate various screenings protocols [62], [71]. In our host-pathogen infection model, most first line anti-tubercular drugs are efficient. Isoniazid (INH), ethionamide and ethambutol were active at 30 µM and efficiently blocked intracellular mycobacterial growth, only pyrazinamide was not active (Figure 2A). A rifamycin family derivate, rifabutin, was the most potent antibiotic in our infection assay with an MIC around 0.25 µM (Figure 2B). We also confirmed that rifabutin curing of an *M. marinum* infection occurred in a dose-dependent manner, and was consistent with confocal microscopy observations of infected cells (Figure 2B and C). At three days post infection, the intracellular bacteria load drastically diminished in presence of rifabutin, as reported by the total fluorescence intensity of bacteria inside infected cells (Figure 1). It is notable that rifabutin action on infection also restored host cell growth in a dose-dependent manner, as judged by the host cell density in Figure 2C. Finally, we assayed a panel of known specific anti-tubercular compounds and broad-spectrum antiobics to treat another intracellular pathogen infection, *L. pneumophila,* in the *A. castellanii* host (Figure 3A). Despite the common ability of these pathogens to avoid phago-lysosomal fusion, the *Legionella*-containing vacuole (LCV) differs from the *M. marinum* phagosome-derived compartment, and therefore, as expected, most of the anti-tubercular antibiotics have no effect on *L. pneumophila* replication. Only drugs such as floxacins [72] and rifampin [73], already reported to be active against *L. pneumophila,* cure *A.castellanii* infected cells.

Because amoebae naturally graze on most innoccuous bacteria but fail to grow on pathogenic bacteria [74], this discriminating ability can be used in an alternative screening assay to test compounds’ ability to restore amoeba growth on a mixture of pathogenic mycobacteria with non-pathogenic *Klebsiella pneumoniae* [75]. As shown in figure 3B, even though this assay is conceptually different from the GFP-based detection assay, it also detects specific anti-mycobacterial antibiotics. However, broad-spectrum antibiotics, such as streptomycin or high concentrations of an anti-tubercular such as isoniazid, eradicate all bacteria and therefore, do not allow *D. discoideum* to generate phagocytic plaques.

### Assay Suitability for Drug Screening and Data Analysis

**Figure 4:**
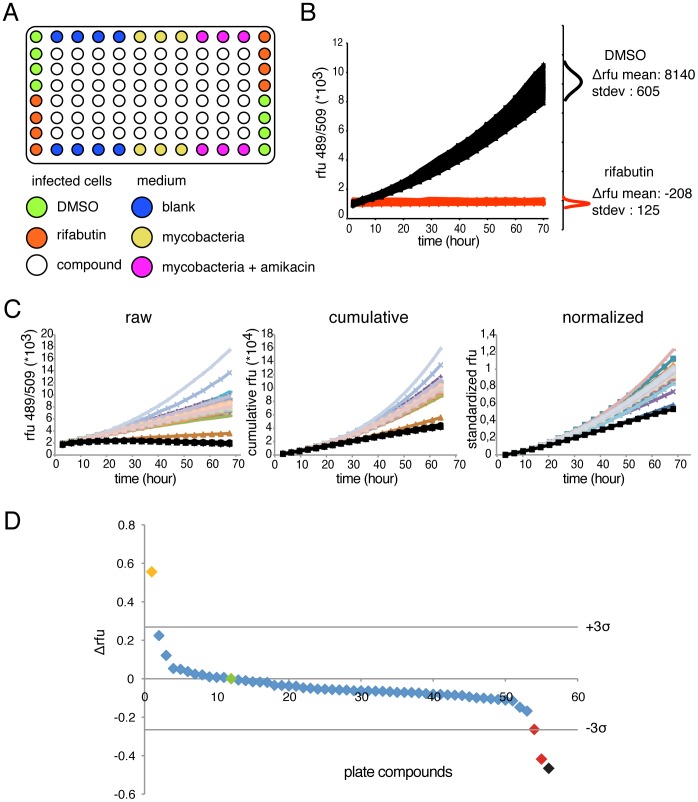
Screening protocol for anti-tubercular compounds. **A.** Scheme of the plate design in 96-well format for compounds screening. **B. Left,** ***representative experiment of*** intracellular growth kinetic of GFP-expressing *M. marinum* measured by fluorescence intensity obtained from one control 96-well plates in presence of DMSO (N = 48) or 10 µM of rifabutin (N = 48). The small graphs on the right represent the normal distribution of the fluorescence difference between 2 and 72 hours of infection for DMSO and rifabutin. Consequently, the Z factor of this experiment was 0.74. **C.** For the statistical analysis, the data are treated in three steps. First, from the ***raw*** data, the value at 68 hours is subtracted from all others and a ***cumulative fluorescence*** curve is drawn. Then, the curves are ***normalized*** to the DMSO standard control. Graphs are representative of one experimental screening plate. **D.** Differential fluorescence values obtained from one experimental plate with diverse compounds are plotted. Horizontal bars represent 3-fold standard deviation from the DMSO mean. The green dot corresponds to the normalized DMSO controls; the orange dot to a putative pro-infectious compound; the black dot to the average of the rifabutin controls; the two red dots to two putative anti-infective compounds. The graph is representative of one experimental screening plate.

Next, we sought to adapt the GFP-based intracellular growth assay for medium-throughput analysis using 96-well plates. Border wells are dedicated to controls needed to test bacteria fitness and amikacin efficiency (Figure 4A). As rifabutin efficiently cures the infection, we decided to include it in the experimental plate design as positive control. Wells containing DMSO (at 1‰, as compound carrier) and rifabutin (10 µM) are used as negative and positive controls, respectively. Therefore, 64 compounds can be tested per 96-well plate. We next measured the quality of our screening protocol to detect potential hits by calculating a Z factor that is a usual parameter to assess screen robustness [76]. From a single standard infection, cells were dispensed in 96-wells, half containing DMSO and half containing 10 µM rifabutin, as shown in figure 4B. The assay sensitivity is high as both controls are clearly separated and, as attested by the standard deviation of the mean, variability between identical wells is low. In both cases, the end-point fluorescence values at 3 days post infection (DPI) were normally distributed. Overall assay robustness is attested by a Z factor score of 0.74 that is excellent for an *‘in vivo’* biological assay, and allows to initiate compounds screening with reasonable confidence.

To allow direct comparison from several plates and multiple infection rounds, raw fluorescence kinetic data (figure 4C, left curve) were transformed. First, to integrate the entire history of the growth kinetics, cumulative curves were built rather than simply using the endpoint measurements were built, and the first point was standardized to 0 by subtracting the value at time zero from each data point (Figure 4C, middle) were transformed as follows. Second, the last value point was standardized to 1 for the mean value of the DMSO controls (Figure 4C, right). Next, normalized cumulative values were ranked relative to the increase or decrease of bacterial growth compared to the DMSO. Representative results from a single experimental plate are presented in figure 4D. The plot represents the difference between the DMSO mean value and each compound of interest. The significance of a compound’s effect was first statistically assessed by its difference from the DMSO control, a difference of more than two or three standard deviations of the DMSO mean being considered significant. Moreover, the strength of inhibition or promotion of bacterial growth was measured by its relative normalized score compared to 1 (DMSO).

### Workflow and Secondary Assays

**Figure 5:**
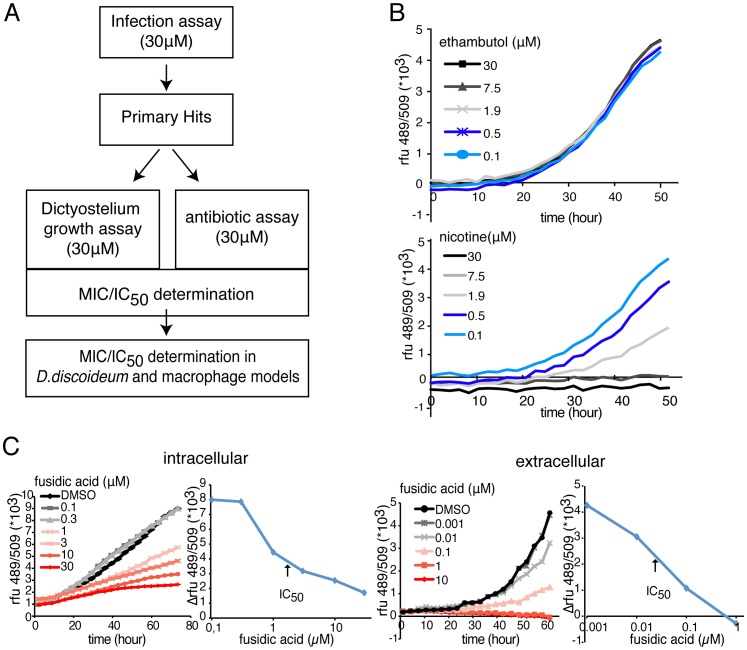
Secondary assays for identifying compounds properties. **A.** Basic scheme of screen workflow including the growth inhibition/cytotoxicity assay and the antibiotic assay before hit validation in other host models system. **B.** Growth inhibition/cytotoxicity assay. Growth kinetics of GFP-expressing *D. discoideum* AX2 cells measured by fluorescence intensity, in the presence of various concentrations of ethambutol (top) or nicotin (bottom). **C.** antibiotic assay**. Left,** intracellular and **Right,** intracellular growth kinetic of GFP-expressing *M. marinum* measured by fluorescence intensity in the presence of various concentrations of fusidic acid, accompanied by the corresponding graphs for IC_50_ determination. B and C are representative experiments from a series with similar outcome.

In order to successfully conduct a screen to identify potential compounds of interest in a complex biological assay, secondary assays are often established and implemented prior to further structure activity relationship studies. In our specific assay setting, two further and major pieces of information can be easily extracted: compound toxicity to the host alone, and antibiotic effect on the bacterium *in vitro*. A basic representation of the screen workflow is presented in figure 5A. The effect of the hit compounds on the host growth and health was tested in the plate reader format with a *D. discoideum* strain expressing a fluorescent reporter, GFP-ABD [77]. For example, we monitored the growth kinetic of this strain in the presence of a classical anti-tubercular antibiotic, ethambutol, and a toxic compound, nicotin (Figure 5B). Ethambutol produced no detectable effect on *D. discoideum* growth, as also observed with most classical antibiotics tested so far, whereas nicotin affects cell growth in a dose-dependent manner (Figure 5B).

A similar assay was used to test the antibiotic activity of the hit compounds directly on *M. marinum* in its standard culture broth (7H9). For example, fusidic acid, a second line antibiotic used for tuberculosis treatment was tested at various concentrations on extracellular and intracellular *M. marinum,* (Figure 5C). In both cases a half inhibitory concentration (IC_50_) can be calculated. IC_50_ values obtained with a small collection of classical anti-tubercular antibiotics are presented in Table 1. Overall, the data highlight a shielding effect of the amoeba host, protecting the mycobacteria from the antibiotics, as previously demonstrated using *M. avium* [59]. In our assay, only rifabutin showed a better effect when tested as anti-infective *in vivo* rather than as antibiotic *in vitro*. This phenomenon is probably explained by its higher liposolubility, which likely enhanced membrane permeability and consequently increased its concentration inside the host cell [78].

**Table 1:** Inhibitory concentrations 50% (IC_50_) values for *M. marinum* growth in extracellular and intracellular conditions.

antibiotic	extracellular IC50 (µM)	intracellular IC50 (µM)
streptomycin	0.1–1	3–7
fusidic acid	0.01–0.1	3–5
ethionamide	0.1–1	3–10
isoniazid	5–10	20–30
rifampin	0.1–0.5	3–10
rifabutin	2–3	1–3
ethambutol	5–10	5–10
levofloxacin	10–30	>30
moxifloxacin	5–10	>30
thioridazine	1–5	5–10
PA-824	3–5	5–10

As presented in Figure 5C, GFP-expressing *M. marinum* growth kinetics were measured in broth culture and during cellular infection in presence of antibiotics in a concentration range, and IC_50_ values were graphically determined from a series of experiments with similar outcome.

## Discussion

In the present study, we detail a screening method to detect anti-tubercular compounds acting in the context of infected amoeba host cells. We first developed and validated suitable conditions for medium throughput screening (MTS). We used fluorescent mycobacteria to monitor intracellular growth in real time by recording fluorescence increase in a 96-well microplate reader. So far, the use of fluorescent mycobacteria is a consensus to avoid the counting of colony forming units, which is hardly compatible with MTS procedures, and allow a fast and reliable way to measure bacterial growth. However, some obvious limitations are due to this detection method. There is no direct way to known how healthy the remaining bacteria are, whose fluorescence remains stable over time. Presently, only a clear decrease in fluorescence intensity, as observed with rifabutin, reflects of a probable bactericidal effect. The long half-life of GFP and the resistance of mycobacteria to cell lysis conferred by their particularly thick cell wall partly explain this phenomenon. Moreover, with this readout, compounds that interfere with cell metabolism, expression of the reporter, or quench its fluorescence can be identified as false positives.

Additionally, introducing host cell-specific fluorescent markers allows the dissection of compound effects on various cell processes like phago-lysosomal trafficking, autophagy induction or lipid storage. Altogether, these fluorescent cell-based approaches can easily be adapted to monitor multiples parameters in a high-content microscopy approach [38]. Therefore, integration of these secondary readouts can provide preliminary knowledge about the mode of action of a compound [39].

As expected, the initial conditions of the infection largely influence the kinetics of bacterial growth. Multiple parameters (percentage of infected cells, cell density, host and bacteria physiological state) modulate the final apparent increase over almost a log range (data not shown). We clearly observe that exponentially growing host cells facilitate bacterial expansion, whereas high confluence partially suppresses it. These findings suggest that cell-to-cell transmission, as observed in *D. discoideum,* is also a significant parameter in *A. castellanii* infections [47].

Surprisingly, depending on the initial bacterial load, the mycobacteria infection leads to host cell death between two to five DPI. Significant cytotoxicity towards the host cell has not been reported during *D. discoideum* infection [79], but was previously noted in macrophages infected by *M. tuberculosis* and *M. marinum* at high MOI and under conditions where phagosomal rupture occurred [69], [80]. Modulation of apoptotic and necrotic cell death has been reported in macrophages infected with virulent and avirulent strains of *M. tuberculosis* [81]. However, in the absence of caspase-dependent apoptotic pathways in amoebae, infection by *M. marinum* likely leads to the necrotic and/or autophagic cell death, mechanistically perhaps similar to the pathways documented in the model amoeba *D. discoideum* [82].

IC_50_ determination of antibiotics in extracellular conditions and within the amoeba host emphasizes the shielding role of *A. castellanii,* as previously shown for *M. avium* [59]. This capacity of *A. castellanii* to protect against antibiotics may be due to a lower membrane permeability and/or enhanced host efflux pump mechanisms that purge intracellular drugs. This observation implies that our assay operates at high stringency to select hit compounds with low IC_50_. Even if direct comparison of anti-tubercular intracellular IC_50_ obtained in various studies is difficult, because of the differences in conditions used (MOI, time resolution), bacterial strains (*M. tuberculosis*, BCG, *M. marinum*) and host (macrophages, amoeba, zebrafish, *D. melanogaster*), the IC_50_ we calculate for our assay reveal a similar shielding effect as seen for other mycobacteria such as *M. avium* during infection in *A. castellanii* [59].

In this study, we reported successful activity of most known anti-tubercular antibiotics to eradicate or attenuate intracellular *M. marinum* growth, with the exception of pyrazinamide. It is known that a low acidic pH is needed to convert pyrazinamide into an active drug, pyrazinoic acid [83], and so far, no data has been published about the pH inside the mycobacteria-containing compartment in amoebae. As discussed in a study reporting about the *D. melanogaster/M. marinum* infection model [84], a lack of vacuole acidification could lead to a poor conversion of the pro-drug into its active form, and thus might explain the inefficiency of pyrazinamide in our system. In addition, a recent paper Ahmad *et al* [85] showed that the minimum inhibitory concentration of pyrazinamide against *M tuberculosis* and *M. bovis* is high. Finally, several Mycobacteria strains such as *M. canettii* are naturally resistant to pyrazinamide [86], as well as clinical isolates of *M. kansasii* and *M. marinum* [87]. Therefore, a higher concentration of pyrazinamide could be needed to impact on *M. marinum* replication in our system.

From our standard experimental data, we calculated a Z factor that lies between 0.6 and 0.8, values that are commonly accepted as highlighting the robustness of an assay [76]. Moreover, we also present here the flow of statistical data analysis that guides us to identify primary hits. Transformations of the raw kinetic data are made to take into account the global history of the growth curve and not only the difference between the values at the first and last time points. This method allows for the robust detection of both anti-infective and pro-infective molecules. Finally, our workflow comprises two secondary assays. A growth inhibition assay using fluorescent *D. discoideum* is used to determine an IC_50_ for negative effects on the host. This allows then to estimate a therapeutic window between effects on the intracellular bacteria and on its host. Yet, the data obtained for amoebae have to be validated for mammalian host cells, e.g. macrophages. The antibiotic assay performed on extracellular bacteria is used to quantitate the difference in efficacy between the effect of the compound on extracellular versus intracellular bacteria, which will permit to identify compounds with most relevant anti-infective activities, being anti-virulence or host defence-boosting mode of action.

Overall, we detailed the establishment of an MTS pipeline for an ‘*in vivo*’ anti-tubercular screen allowing to test in moderate turnover time, 64 molecules per 96-well plates, with excellent sensitivity and good anti-mycobacterial specificity. Finally, we speculate that the use of amoebae hosts, which are natural vectors for many bacteria and have already proven powerful in the elucidation of mechanisms underlying host-pathogen relationships [47], [79], also represent a worthy alternative model to contribute to hit and lead identification and drug development to fight tuberculosis.

## Materials and Methods

### Bacteria and Cell Cultures

*Acanthamoeba castellanii* (ATCC 30234) was grown in PYG medium at 25°C as described (Moffat and Tompkins, 1992; Segal and Shuman, 1999) using proteose peptone (Becton Dickinson Biosciences) and yeast extract (Difco). The *D. discoideum* strain expressing GFP-ABD [71] was grown in HL5c medium at 22°C. Mycobacteria, the *M. marinum* M-strain (wild-type), the L1D mutant [65] and *M. smegmatis* (generous gift from Gareth Griffiths) were cultured in Middlebrook 7H9 (Difco) supplemented with 10% OADC (Becton Dickinson), 5% glycerol and 0.2% Tween80 (Sigma Aldrich) at 32°C in shaking culture. *M. marinum* and the msp12::GFP plasmid were gifts from Dr. L. Ramakrishnan (Washington University, Seattle, USA). The *M. marinum* strain expressing GFP in a constitutive manner was obtained by transformation with msp12::GFP, and cultivated in the presence of 20 µg/ml kanamycin.

### Acanthamoeba Infection Assay

*A. castellanii* were cultured in PYG medium in 10 cm Petri dishes at 25°C, and passaged the day prior to infection to reach 90% confluency. *M. marinum* were cultivated in a shaking culture at 32°C to an OD_600_ of 0.8–1 in 7H9 medium. Mycobacteria were centrifuged onto a monolayer of *Acanthamoeba* cells at an MOI of 10 to promote efficient and synchronous uptake. Centrifugation was performed at RT at 500 g for two periods of 10 min. After an additional 20–30 min incubation, extracellular bacteria were washed off with PYG and infected cells were resuspended in PYG containing 10 µM amikacin. 5×10^4^ infected cells were transferred to each well of a 96-well plate (Cell Carrier, black, transparent bottom from Perkin-Elmer) with preplated compounds and controls. The course of infection at 25°C was monitored by measuring fluorescence in a plate reader (Synergy H1, BioTek) for 72 hours with time points taken every 3 hours. Time courses were plotted and data from all time points were used to determine the effect of compounds versus vehicle controls. To take into account possible autofluorescence of the compounds, RFU data of the first time point were subtracted from all time points. Cumulative curves were calculated. The activities of the compounds were determined by analysing maximum difference of compound cumulative curve to the 12–16 vehicle controls.

### Fluorescence Microscopy

*Acanthamoeba* cells were infected with GFP-expressing *M. marinum* as described above. Infected cells were monitored in 96-well plates (Cell Carrier, black, transparent bottom from Perkin-Elmer). Recordings were performed using a Leica LF6000LX microscope (100x 1.4 NA oil immersion objective).

### Phase Contrast Microscopy

*Acanthamoeba* cells were infected with GFP-expressing *M. marinum* as described above. Infected cells were monitored in 96-well plates (Cell Carrier, black, transparent bottom from Perkin-Elmer). Recordings were performed using a CKX41 inverted microscope.

### Antibiotic Activity Assay

10^4^ GFP-ABD-expressing *D. discoideum* cells were transferred to each well of 96-well plates allowed to attach for 20–30 min. Cell growth at 25°C was monitored by measuring the GFP fluorescence in a fluorescent plate reader (Synergy H1, company) for 72 hours with a time point taken every 3 hours.

### Growth Inhibition Assay

10^5^ GFP-expressing *M. marinum* were transferred to each well of 96-well plates. Bacterial growth at 25°C was monitored by measuring the GFP fluorescence in a fluorescent platereader (Synergy H1) for 72 hours with a time point taken every 3 hours.

### Statistical Analysis

The Z factor was calculated using the means and standard deviations of both positive and negative controls (µ_p_, σ_p_ and µ_n_, σ_n_). The following formula was applied: Z-factor = 1–3(σ_p_+ σ_n_)/|µ_p_-µ_n_|.

### Intracellular Replication of *L. pneumophila*

*A. castellanii* amoebae were cultured in PYG medium and passaged the day prior to infection such that 2×10^4^ cells were present in each well of a 96-well plate (Cell Carrier, black, transparent bottom from Perkin-Elmer). Cultures of *L. pneumophila* harbouring the GFP-producing plasmid pNT-28 were resuspended from plates to a starting OD_600_ of 0.1 in AYE medium, and grown overnight on a rotating wheel at 37°C to an OD_600_ of 3. Bacteria were diluted in LoFlo medium (ForMedium) such that each well contained 8×10^5^ bacteria, an MOI of 20. Infections were synchronised by centrifugation at 1500 rpm for 10 minutes. Infected cultures were incubated in a 30°C incubator and the GFP fluorescence was measured by a plate spectrophotometer at appropriate intervals (Optima FluoStar, BMG Labtech) [88]. Time courses were constructed and data from the point directly after entry up to stationary phase were used to determine the effect of compounds versus vehicle control.

### *Dictyostelium* Growth on a Bacteria Lawn

Because *D. discoideum* cannot grow on lawns of virulent *M. marinum*, a specific growth assay was developed [89]. It consists in resuspending a pellet from one volume of centrifuged mid-log phase mycobacterial cultures in an equal volume of an overnight culture of *K. pneumoniae* diluted 10^5^ fold. Then, 50 µl aliquots of the bacterial suspension were plated on 2 mL plugs of solid SM+Glucose agar medium in a 24-well plate format and left to dry for 2–3 hours. Finally, 1,000 *D. discoideum* cells were added on top of the bacterial lawn. Plates were incubated for 5–9 days at 25°C and the formation of phagocytic plaques was monitored and quantified.
